# Large Scale Application of Vibration Sensors for Fan Monitoring at Commercial Layer Hen Houses

**DOI:** 10.3390/s101211590

**Published:** 2010-12-16

**Authors:** Yan Chen, Ji-Qin Ni, Claude A. Diehl, Albert J. Heber, Bill W. Bogan, Li-Long Chai

**Affiliations:** Department of Agricultural and Biological Engineering, Purdue University, 225 S. University St., West Lafayette, IN 47907, USA; E-Mails: yanchenpurdue@gmail.com (Y.C.); diehl@purdue.edu (C.A.D.); heber@purdue.edu (A.J.H.); bogan@purdue.edu (B.W.B.); lchaipurdue@gmail.com (L.L.C.)

**Keywords:** air quality, animal agriculture, emission, measurement, MEMS, ventilation rate

## Abstract

Continuously monitoring the operation of each individual fan can significantly improve the measurement quality of aerial pollutant emissions from animal buildings that have a large number of fans. To monitor the fan operation by detecting the fan vibration is a relatively new technique. A low-cost electronic vibration sensor was developed and commercialized. However, its large scale application has not yet been evaluated. This paper presents long-term performance results of this vibration sensor at two large commercial layer houses. Vibration sensors were installed on 164 fans of 130 cm diameter to continuously monitor the fan on/off status for two years. The performance of the vibration sensors was compared with fan rotational speed (FRS) sensors. The vibration sensors exhibited quick response and high sensitivity to fan operations and therefore satisfied the general requirements of air quality research. The study proved that detecting fan vibration was an effective method to monitor the on/off status of a large number of single-speed fans. The vibration sensor itself was $2 more expensive than a magnetic proximity FRS sensor but the overall cost including installation and data acquisition hardware was $77 less expensive than the FRS sensor. A total of nine vibration sensors failed during the study and the failure rate was related to the batches of product. A few sensors also exhibited unsteady sensitivity. As a new product, the quality of the sensor should be improved to make it more reliable and acceptable.

## 1. Introduction

Air pollution has become one of the most significant environmental concerns in sustainable agriculture development. Excess emissions of agricultural pollutants can result in environmental pollution [1] and ecological damage near the facilities, and contribute to climate change on a global scale [2]. High pollutant concentrations above exposure thresholds inside confined spaces can also cause health related problems, or even deaths of animals and workers [3].

Researchers began to experimentally study agricultural air quality (AAQ) in the 1950s, with a two-day measurement of ammonia (NH_3_) concentration in a broiler house [4]. In the early days, the types of pollutants monitored for AAQ were limited, and the measurements were short-term. For example, ammonia concentration was studied for 24 h in two dairy barns [5]; and hydrogen sulfide (H_2_S) emission was measured for 10 d in swine buildings [6]. With the development of measurement technology and the increasing awareness of AAQ, more pollutants were studied and monitoring periods became much longer [7]. The agricultural air pollutants that have been studied in animal buildings include NH_3_, H_2_S, carbon dioxide (CO_2_), methane (CH_4_), non-methane hydrocarbons (NMHC), volatile organic compounds (VOCs), particulate matter (PM), and odor. Intensive studies with continuous aerial pollutant concentration and emission monitoring lasted from several months [8] to 2 years [9]. To establish reliable emission factors for agricultural air pollutants from typical animal buildings, a long-term National Air Emission Monitoring Study (NAEMS) started in 2007 and was completed in 2010 [10].

To accurately determine air pollutant emission rates from an animal building, continuous measurement of ventilation rate and pollutant concentration are both imperative, because the air pollutant emission rate is the product of ventilation rate and pollutant concentration. The concentrations of pollutants can usually be analyzed off-site by collecting samples from animal buildings with subsequent laboratory analysis, or determined on-site and real-time by using gas analyzers or sensors. Although pollutant sampling and concentration measurement has a common issue with quality assurance and quality control [11], ventilation measurement appears to be more technically challenging. As a consequence, ventilation rate measurement typically introduces the greatest uncertainties in air pollution emission rate estimations [12].

The current methods for ventilation rate measurement varied in different studies at different types of animal buildings. There are three continuous monitoring methods available for the mechanically ventilated animal buildings with large numbers of ventilation fans. They include fan rotation methods, airspeed measurement methods, and fan indication methods [13]. The fan rotational speed method employed the fan rotational speed (FRS) sensor, which is used to monitor the fan blade rotational speed. However, the cost of the FRS sensor and its data acquisition hardware is relatively high and its large scale application is expensive. The fan air speed measurement method utilizes small impeller anemometers for continuously measuring the air velocity at a fixed point in the fan outlet. Nevertheless, the price of the anemometer is even higher. The fan indication method employs sail switches, vibration sensors, contact relays, and other devices to monitor fan on/off status. These devices are relative low priced and suitable for large scale applications. However, the sail switch is susceptible to mechanical failure and sensitive to dust build-up. Vibration sensors were introduced to fan on/off status monitoring in 2005 [14]. The principles of the vibration sensor measurement were illustrated by Ni *et al.* [14] and Darr *et al.* [15].

Fan on/off status represents the fan operation and can be used to record the operation time of individual fans. The recorded data are valuable in estimating fan airflow rates to improve pollutant emission rate calculation. Fan on/off data can be converted to airflow rate by using fan performance models and static pressures. The fan performance model describes the mathematical relationship between fan airflow rate and the static pressure that the fan has to overcome. It can be obtained in controlled laboratory conditions and verified on-site using a portable fan testing device or the traverse method. On-site fan test is necessary because fan capacity can drop from 12% to 55% between published and as-found airflow rates at field conditions due to fan degradation [16].

The principle of fan on/off status monitoring is based on some special physical and electrical phenomena between operating and stopping of the fan. Fan on/off monitoring using vibration sensors has several advantages, including low cost, easy installation, small size, real-time monitoring, and freedom from dust and wind interference [14]. These advantages demonstrated the potential for its large scale application.

At a NAEMS layer hen monitoring site, vibration sensors were used to individually monitor 164 fans for two years. The objective of this work was to study this case of vibration sensor application. Specifically, this study aimed to:

1. describe the large scale application of the new vibration sensor in long-term air quality research,
2. compare the reliability and accuracy of the vibration sensor with the FRS sensor, and
3. evaluate the applicability and future improvement of the sensor.

## 2. Materials and Method

### 2.1. Test Site

**Figure 1.**
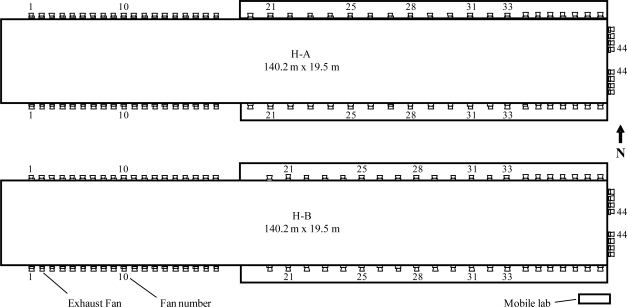
Floor plan of the layer houses (H-A and H-B) and the distribution of ventilation exhaust fans. Numbered fans are variable-speed except for fans 44 in both houses.

The monitoring site was located in northeast Indiana and was one of 15 barn monitoring sites in the NAEMS. This site consisted of an egg-processing plant, two high-rise caged-hen houses, eight manure-belt caged-hen layer houses, two cage-free laying houses, and one free standing manure shed [17]. This study was conducted in the two manure-belt caged-hen layer houses, denoted here as H-A and H-B (Figure 1).

Each house was 140.2 m long and 19.5 m wide and had 14 variable-speed and 74 single-speed wall fans of 130 cm diameter (VX511F3CR, Aerotech Inc., Mason, Mich.). The two houses were almost identical except for the spatial distribution of Fans 20 to 33. The rotational speeds of the seven variable-speed fans (Fans 1, 10, 21, 25, 28, 31, and 33) in each side wall were controlled by a single controller. All the variable-speed fans operated continuously.

The single-speed fans in each house were grouped into 12 stages, each of which consisted of 4 to 12 fans. Fans in the same stage were spatially distributed as uniformly as possible in the house and operated simultaneously. The fan control systems in the houses were temperature-based. They automatically turned on more stages of fans to provide better cooling when the in-house temperature was higher, so that the fans operated only when needed.

### 2.2. Vibration Sensor and Fan Monitoring

**Figure 2.**
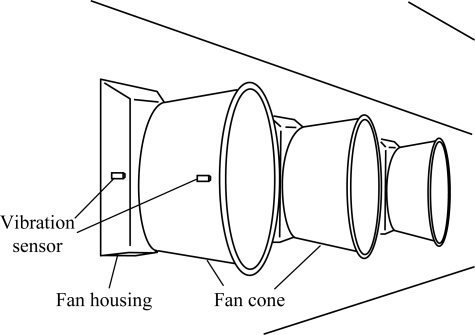
Mounting locations of vibration sensor on the fan housing or fan cone [14].

A total of 164 vibration sensors (Model OSU-06, Ohio State University, Columbus, Ohio) were installed for fan on/off status monitoring in the two houses. The 148 single-speed fans were all monitored with vibration sensors. The FRS of the 28 variable-speed fans were monitored with magnetic proximity FRS sensors (Model Cherry MP100701, Cherry Corporation, Pleasant Prairie, Wisc.) to provide real-time data for the rotational speed of fan blades. Sixteen of the 28 variable-speed fans were also monitored with vibration sensors. The vibration sensor used in this study was the integration of an embeddable accelerometer (ADXL320, Analog Devices, Norwood, Mass.), which was also known as micro-electro-mechanical systems (MEMS), and a single conditional circuit including a decouple capacitor, a diode array, a resistor-capacitor filter, an operational amplifier (MAX495, Maxim Integrated Products, Sunnyvale, Cal.), and a Schmitt trigger circuit (SN74LVC2G14, Texas Instruments, Dallas, Texas) [15]. The sensors were installed on the housing or cone of each ventilation fan (Figure 2) in October, 2007 and continuously operated for 24 months.

### 2.3. Performance Assessment of Vibration Sensor

Data from all on-line instruments and sensors at the measurement site, including the vibration and FRS sensors, were acquired by an on-site computer system (OSCS) in the mobile lab (Figure 1) and synchronized, and saved every 15 and 60 s in two separate data files [18]. Both types of data files were used for data analysis. The vibration sensor outputs were “on” and “off” binary signals. The OSCS converted the signals to percent of time (% t) that the sensor was “on”. The FRS sensor output was in revolutions per minute (rpm).

The performance of the vibration sensors were assessed at the variable-speed fans so that the sensors could be tested at conditions of different fan vibration frequencies and magnitudes due to the variation of fan speeds. In addition, the FRS sensors installed in the variable-speed fans provided reference signals to be compared with the responses of the vibration sensors.

To assess the measurement accuracy, which is a qualitative term [19], the maximum error, average error, and standard deviation of the vibration sensor measurements errors were calculated by comparing the measurement data (% t) from the vibration sensors with the on/off data (% t) converted from FRS data (rpm). The conversion criteria are described in Table 1. A fan full-time operation threshold of 200 rpm was chosen based on experimental data.

The average error (E_a_) was calculated with Equation 1:

(1) $${\text{E}}_{a}=\frac{\sum_{i=1}^{\text{N}}\left({\text{t}}_{\text{i},\text{Vb}}-{\text{t}}_{\text{i},\text{FRS}}\right)}{\text{N}}$$

Where:

N = number of measurement points (dimensionless)
t_i,FRS_ = converted operation time based on FRS sensor measurement at *i^th^* point (0–100% t)
t_i,Vb_ = percentage of fan operation time measured by vibration sensor at *i^th^* point (0–100% t)

The standard deviation (*s*) of the error (SDE) was calculated with Equation 2:

(2) $$s=\sqrt{\frac{\sum_{i=1}^{\text{N}}{\left[({\text{t}}_{\text{i},\text{Vb}}-{\text{t}}_{\text{i},\text{FRS}})-{\text{E}}_{a}\right]}^{2}}{\text{N}-1}}$$

Comparison between the vibration sensors and the FRS sensors was used to analyze the vibration sensor performances including measurement errors and response time, because the FRS sensors had higher time resolution in their digital output signals. Based on the conversion criteria in Table 1, eight paired vibration and FRS sensors were compared, with 18.3 h of measurement for each pair (4,400 data points of 15-s each).

To study the cases of vibration sensor failure and the environmental effects on the sensor signals, data obtained from different sensors on the same fan stage were analyzed. Sensor failure was identified when signals from several sensors on fans of the same stage did not match with each other.

## 3. Result and Discussion

### 3.1. Effectiveness of Fan Monitoring

**Figure 3.**
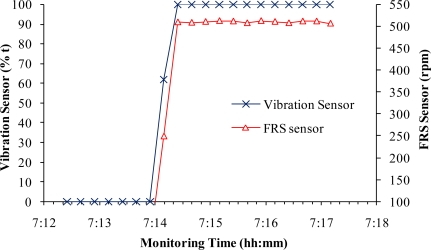
Comparison of responses between a vibration sensor and a FRS sensor of the same fan.

**Figure 4.**
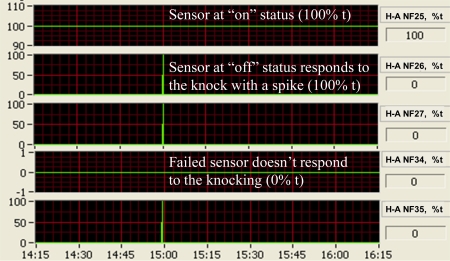
On-screen vibration sensor signals responding to fan cone knocking during sensor operational condition inspection.

Figure 3 presents an example showing the response of a vibration sensor to the fan start-up and continuous operation compared with an FRS sensor, which was installed on the same fan as the vibration sensor. The responses of the two sensors matched well in the data saved every 15 s. This fast response could generally meet the demands of air quality studies at animal houses because most of the data recording time was every min or longer in reported comprehensive air quality studies [4,7]. The real-time response of the vibration sensor was shown to be much faster during the sensor operational inspection when the fan cones were slightly knocked to manually create a vibration (Figure 4).

If the fan was off and the sensor responded to the knocking normally, a sensor signal spike was seen immediately in the real-time 1 Hz signal charts in the OSCS [18]. No spikes during the knocking indicated certain problems associated with the sensor. In addition, the vibration sensor had a build-in LED that emitted a red light when it detected vibration. This made the on-site sensor inspection more convenient because the sensors were installed 10 to 200 m away from the mobile lab. The sensitive response demonstrated that the vibration sensors could provide effective monitoring for ventilation fan operations.

### 3.2. Measurement Error

The average error and SDE analyzed from the eight pairs of 18.3-h data illustrated that the measurement errors introduced by the vibration sensors could be divided into two groups (Table 2):

1. Continuous fan operation. The average error and SDE for each paired comparison was equal to zero when the fan was in continuous operation (no on/off switch).
2. Non-continuous fan operation. The average error and SDE for each paired comparison was close to zero and negligible when the fan was switched on and off during the tests.

Compared with the FRS sensors, the variation of SDEs at different on/off switch times, as shown in the first four rows in Table 3, signified that the vibration sensor measurement errors were associated with the frequency of on/off switching during fan operation. The SDE obtained for the paired data with six switch times were higher than the data with four switch times. This was because the vibration sensor measurement error for a sensor in normal operational condition could only occur when the fan was switched between “on” and “off”.

### 3.3. Interference by Thunderstorm

**Figure 5.**
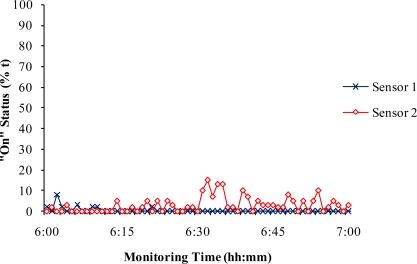
Recorded 1-min data showing the vibration sensor signals affected by extremely heavy rain during a thunderstorm.

During the 2-year field application, there was one recorded observation of interference on the measurement of vibration sensors by extremely heavy rain during a thunderstorm. The heavy rain drops striking the fan cone generated vibration signals. These signals were clearly seen in the computer monitor as spikes similar to the ones shown in Figure 4, but with short durations. When the sensor signals were averaged at 1-min integration time and saved in data files, the data showed that the signals from sensors 1 and 2 had some spikes between 0% and 20% operation time (Figure 5).

This information recorded in the 1-min data file could be interpreted as if the fans were turned on and off quickly. However, the ventilation fans were not controlled to be switched “on” and “off” within a 1-min period. Therefore, these errors that were introduced by heavy rains could be easily identified and filtered during post-measurement data processing. No other interferences that introduced signal errors were noticed during the field study.

### 3.4. Sensor Failure

A total of nine out of the 164 installed vibration sensors failed, causing them to completely unresponsive to fan operations, and were replaced during the 2-year study. The average sensor failure rate was 5.5% (Table 3).

Data from the field application indicated that the sensor failure rates were related to the different batches of vibration sensors during manufacturing. In the field setup, the vibration sensors were received in three batches. Table 3 shows that the first batch had the highest failure rate (13.9%), the second batch was better (5.6%), and the third batch was the best (1.4%).

Recorded data demonstrated that a few vibration sensors suddenly stopped responding to fan vibrations after a normal operational period. This type of sensor problem apparently was associated with the failure of electronic components inside the sensor. Because the sensor was at initial production stage and the three batches were manufactured on different dates, the variation of failure rates among the batches might be associated with the sensor manufacturing quality. Table 3 demonstrated that, as a new product, the quality of the sensor can be potentially improved to achieve higher reliability that is extremely important for its future successful application.

### 3.5. Unsteady Sensitivity

**Figure 6.**
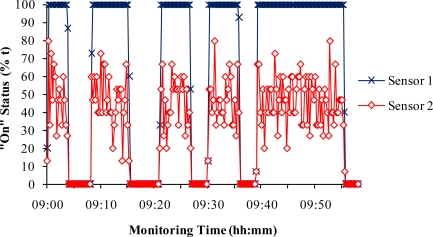
Comparison of a normal sensor (Sensor 1) and a sensor with unsteady sensitivity (Sensor 2) shown in the 15-s data.

**Figure 7.**
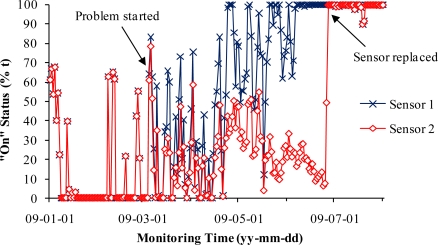
Normal signals from Sensor 1 compared with unsteady sensitivity signals from Sensor 2 using daily mean data from January 1 to August 1, 2009.

**Figure 8.**
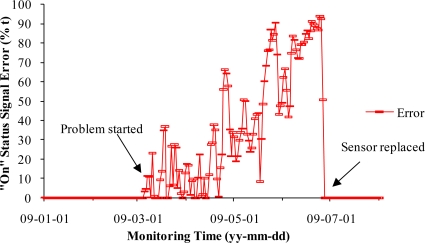
The absolute error between unsteady and normal sensor signals from January 1 to August 1, 2009.

In addition to the complete failure of some sensors, unsteady sensitivity was a problem encountered during the study. Based on the original field report and data analysis, the cases of unsteady sensitivity shared some common features (Figures 6 to 8). Compared with Sensor 1 in Figure 6, Sensor 2 in the same fan stage had significantly fluctuating outputs. Although its outputs were zero when the fan status was “off”, they jumped between “on” and “off” when the fan operated. When the outputs were averaged, the “on” status was lower than 100% as if the fan had been turned on and then off within 15 s (Figure 6).

The problem shown in Figure 6 was caused by the shift of the sensor vibration detection threshold, which could be corrected by manually adjusting the build-in potentiometer in the sensor. However, in long-term and continuous agricultural air quality research, the sensors and equipment were unattended most of the time. Not all the sensor problems could be timely identified and corrected. Therefore, more stable sensitivity of the sensors could have provided higher quality fan monitoring data.

Figure 7 shows that the unsteady sensitivity of Sensor 2 gradually developed from March 5 to June 26, 2009. As a consequence, the signal differences between the normal sensor (Sensor 1) and the failing sensor (Sensor 2) slowly increased (Figure 8).

Sensor 2 was replaced with a new sensor on June 26, 2009. The new sensor restored the signals that agreed well with the normal sensor (Sensor 1). This case demonstrated that the unsteady sensor could start from small errors of 1% and the errors could gradually increase to 100%, leading to eventual failure. This process of failure could last for as long as four months. This type of failure might be the result from a gradual wearing-out of sensor inner mechanism, which manifested time-accumulated decay. There were two vibration sensor failures that matched this case during the two year study. Comparison of signals of different vibration sensors that monitored the same stage fans could help to identify failing sensors, replace them in time, and reduce monitoring errors. However, a vibration sensor with more stable sensitivity will help to improve data quality.

### 3.6. Comparison with FRS and Ventilation Sensors

Cost is always one of the main factors for sensor selection in research projects with limited funding. Compared with a Cherry MP100701 magnetic proximity FRS sensor that cost $30 each in this study for FRS measurement, a vibration sensor was $2 more expensive (Table 4). However, the vibration sensor had only about 10% cost in installation because it was easy to attach to a fan and used much less labor and materials. In addition, it cost only $3 for data acquisition (DAQ) hardware per sensor when using one channel in a digital input device (Model USB-DIO96-H, Measurement Computing Co., Norton, Mass,). As a comparison, the Cherry sensor needed $35 per sensor when using one channel of a counter device (Model USB-4303, Measurement Computing Co.).

Moreover, the PC and Windows operation system for DAQ had limitations on the number of USB DAQ devices. The OSCS in this air quality study that acquired data at 1 Hz could only connect to maximum of six USB-4303 counters, each with 10 FRS sensors [18]. This restricted the total number of FRS sensors that could be used in a monitoring system at layer houses with large number of fans.

Both vibration sensors and FRS sensors demonstrated the quick response and high sensitivity to fan operations needed in fan monitoring in air quality studies. However, the vibration sensors only detected fan on/off status while the FRS sensors provided fan rotational speed information, which is critical when monitoring variable-speed fans.

The method of single-speed fan monitoring by detecting its vibration was proved to be effective and a good option when monitoring large numbers of single-speed ventilation fans in air quality research. The technical issues of this particular vibration sensor, *i.e.*, the sensor failure and unsteady sensitivity, appeared to be related to the manufacturing quality. Future improvement focusing on increasing its reliability for more worry-free applications and lowering its costs will make the vibration sensor a more applicable device for fan monitoring.

The ventilation sensor [20,21], also called airflow transmitter [22] or fan wheel monitor, is currently the most accurate device for continuous ventilation measurement at animal buildings. This sensor originally had an accuracy of 60 m^3^ h^−1^ in a measurement range from 200 to 5,000 m^3^ h^−1^ at pressure difference of 0–120 Pa [20]. The recent models of the commercial sensor have expanded measurement range to 200–20,000 m^3^ h^−1^ but are still limited for fans of <80 cm diameters and are mainly used in Europe [21,22]. Therefore, they are not suitable for measuring ventilation rates at commercial layer houses that are equipped with large numbers of fans of 130 cm diameter in North America. In addition, an 80-cm airflow transmitter costs approximately $1,000, which is too expensive for large scale applications.

### 3.7. Recommendation for Vibration Sensor Application

Theoretically, all fans in the same fan stage at an animal building should operate simultaneously. In reality, however, it often happens randomly that one or more fans do not operate as other fans in the same stage due to fan failure, power problem, maintenance, or other reasons. This introduces ventilation measurement errors if these fans are not individually monitored. Therefore, monitoring all ventilation fans individually and continuously in comprehensive air quality studies is recommended instead of only monitoring selected fans to represent all fans in a stage. This study demonstrated that the low cost and simple data acquisition requirement of the vibration sensors have made the all-fan monitoring economically and technically feasible.

However, it is also recommended that the quality of the vibration sensor be greatly improved to reduce the failure rate. This is essential to increase the confidence level of fan monitoring and make the vibration sensor more acceptable.

## 4. Conclusions

1. The 164 vibration sensors in the two-year study demonstrated their applicability and proved that detecting fan vibration was an effective method to monitor the on/off status of single-speed ventilation fans. However, the vibration sensor was not preferred for variable-speed fan airflow determination.
2. The vibration sensors had quick response and high sensitivity to fan operations and could satisfy general requirements of air quality research. On-site inspections of their operation were convenient.
3. The tested vibration sensors had an average failure rate of 5.5% during the two-year study. Some sensors demonstrated unsteady sensitivities and gradual increases in signal errors that lasted for four months. Comparison of sensor signals from the same stage of fans could help to identify sensor problems for early correction.
4. Interferences (e.g., caused by heavy rain drops) to the vibration sensors could be identified if the sensor signals showed below 20% operation time in the 1-min data. These errors could be filtered and corrected during post-measurement data processing.
5. Although the vibration sensor cost $2 more than the magnetic proximity FRS sensor, its installation and data acquisition hardware cost was $77 lower than the FRS sensor. The vibration sensor was suitable for monitoring large numbers of single-speed fans with reasonable costs.
6. It is recommended that all fans be individually and continuously monitored in comprehensive air quality studies to increase confidence in aerial emission measurement. The vibration sensor was proved to be economically affordable and technically feasible for the all-fan monitoring.
7. As a new product, the technical issues of the vibration sensor, including sensor failure and drifting sensitivity, all appeared to be related to the sensor manufacturing quality that should be improved to increase its reliability.

## Figures and Tables

**Table 1.** Fan rotational speed (FRS) to vibration sensor (Vb) on/off time conversion criteria.

**Measured FRS (rpm)**	**Converted Vb (% t)**
0 < FRS < 200	FRS / 2
200 ≤ FRS	100

**Table 2.** Measurement error comparison of vibration sensors with FRS sensors.

**Sensor location**	**Average error**	**Maximum error**	**SDE**	**Time of on/off switch**	**Operation (% t)**
H-A NF1	0	0	0	0	100
H-A NF10	0	0	0	0	100
H-A NF21	0	0	0	0	100
H-A NF33	0	0	0	0	100
H-B SF31	0.07	14	0.72	4	95.84
H-B SF33	−0.16	12	0.54	4	95.90
H-A SF10	0.05	50	1.28	6	42.53
H-A SF33	0.03	43	0.97	6	42.55

Note: Sensor location NF = north side fan; SF = south side fan; SDE = standard deviation of the error.

**Table 3.** Vibration sensor replacement in the two houses due to sensor failures.

**Batch number**	**Total sensors (n)**	**Failure ratio (%)**	**Failed sensors (n)**	**Sensor location**	**Installed (yy-mm-dd)**	**Replaced (yy-mm-dd)**
1	36	13.9	5	H-B SF22	07-10-09	09-06-26
				H-B SF24	07-10-09	09-06-26
				H-B SF34	07-10-09	08-10-23
				H-B SF40	07-10-09	09-06-26
				H-B SF41	07-10-09	08-01-10
2	54	5.6	3	H-B NF18	07-10-15	08-06-27
				H-B NF24	07-10-15	08-10-23
				H-B NF41	07-10-17	08-01-25
3	74	1.4	1	H-A NF34	07-12-05	09-02-13
Summary	Total 164	Average 5.5	Total 9			

Note: Sensor location NF = north side fan; SF = south side fan.

**Table 4.** Comparison of the vibration sensor with the magnetic proximity FRS sensor.

	**Vibration sensor**	**FRS sensor**
Sensor cost	$32 (OSU-06)	$30 (Cherry MP100701)
Installation cost a	$5	$50
DAQ hardware cost b	$3 (USB DIO-96H)	$35 (USB-4303 counter)
Pros and cons	Easy installation Easy in-field inspection Relatively low cost Suitable for large-scale application Only provide fan on/off status Product quality needs improvement	Provide fan on/off and rotational speed information Suitable for both variable and single-speed fans High accuracy Relatively high cost Restriction for large number of sensors in a single DAQ system

Note:

a Installation cost per sensor was approximate and based on actual cost during this study. It included parts and labor but excluded sensor cable cost.

b Cost per DAQ channel.
